# Early In-Process Prediction of GMA Weld Quality from Laser Doppler Vibrometer Signals

**DOI:** 10.3390/s26165011

**Published:** 2026-08-07

**Authors:** Wojciech Jamrozik, Bernard Wyględacz, Jacek Górka

**Affiliations:** 1 Department of Fundamentals of Machinery Design, Silesian University of Technology, Konarskiego Str. 18a, 44-100 Gliwice, Poland; 2 Department of Welding Engineering, Silesian University of Technology, Konarskiego Str. 18a, 44-100 Gliwice, Poland; bernard.wygledacz@polsl.pl

**Keywords:** gas metal arc welding, GMA, laser Doppler vibrometry, LDV, in-process monitoring, early recognition, streaming classification, weld quality, non-contact sensing

## Abstract

Post-process weld quality assurance detects a defect only after the joint has been completed and therefore cannot intervene while an unstable weld is still being produced. The question addressed here is how much of a seam must be observed before its final quality state can be recognized. This study investigates whether the final quality state of a gas metal arc (GMA) weld can be recognized from the initial segment of the seam using only a laser Doppler vibrometer as the diagnostic sensor. Thirty-two bead-on-plate GMA welds were produced under stable and deliberately destabilized conditions. The welding current channel was used only for offline arc segmentation and independent reference, whereas classification used vibration-derived time, frequency, envelope, and wavelet features extracted in 0.10 s windows. Causal prefix features were evaluated with leave-one-weld-out validation and weld-level bootstrap confidence intervals, and a streaming variant updated the probability of a defective weld window by window. The final process-stability state was distinguishable after approximately 5 mm of seam (AUC = 0.94), with high discrimination through the early-to-mid seam. As an online alarm, the aggregate prefix rule produced no false alarms among six acceptable welds, detected 95% of the defective welds, and achieved a median recognition lead of ~3.6 s. However, these specificity and lead-time estimates are coarse because only six acceptable welds were available, and with a single exception, the defective welds were unstable throughout; thus, the results mainly reflect early recognition of an established state. Decision latency was nearly constant in time (0.15–0.25 s), so the spatial decision position and remaining lead time depended mainly on welding speed. The results support non-contact early recognition of unstable GMA welding, while larger balanced datasets with mid-weld transitions are needed to validate true defect-onset anticipation.

## 1. Introduction

Gas metal arc (GMA) welding is one of the most important joining processes used commercially in industrial applications. It combines high deposition rate and high productivity, is amenable to mechanization, and can be used for steels, as well as non-ferrous alloys. Real-life applications of GMA welding have certain drawbacks, caused by the intrinsic dynamics of the process. The arc, additional molten material, type of droplet transfer, weld pool, and shield atmosphere have to be considered as a coupled thermo-electromagnetic–mechanical system. The stability of this system depends on arc voltage, welding current, wire feed rate, travel speed, and shielding gas composition and rate. Moreover, preparation of joined workpieces is also a crucial factor determining process stability. Fundamental descriptions of these instability phenomena, the criteria used to quantify arc stability, and their dependence on shielding gas are given in [1,2,3]. For short-circuiting and globular metal transfer regimes, even a small deviation from the operating window covering stable operation can influence and change the transfer mode, alter the instantaneous heat input, and generate welding instabilities and even faults (spatter, porosity, undercut, underfilling, lack of fusion, or excessive penetration) [4,5,6,7]. Every type of defect is a real problem for industry, especially in automated production. Because of manufacturing speed and the repeatable nature of automated tasks, unwanted settings or environmental circumstances can propagate over a large batch of produced elements before classical inspections identify the cause.

Industrial weld quality assurance, especially for methods based on electric arc as the heat source, is still dominated by post-process non-destructive testing (NDT) methods. Visual inspection and ultrasonic, radiographic, magnetic-particle, or dye-penetrant methods are indispensable and required for product certification and final acceptance. Although reliable, these methods cannot always be applied to indicate possible defects during the production process, when the bead is formed. It can be stated that classic NDT leads to consequence detection rather than revealing the process conditions responsible for defect generation. Reported systems illustrate this practical advantage. A review of real-time sensing in GMA welding is provided in [5]. Shin et al. [6] used welding voltage signals for real-time porosity detection, while further GMA welding studies applied machine learning analysis [7] and empirical mode decomposition features for quality diagnosis [8]. These approaches identify abnormal conditions from in-process signals rather than only during final inspection. The growing adoption of Industry 4.0 manufacturing has therefore shifted attention toward data-driven in-process monitoring. In this paradigm, the goal is not only to classify a completed joint but also to recognize process instabilities early enough to correct parameters or finish running the process to minimize the possible loss of material and energy. Researchers and equipment producers offer a wide range of solutions and hardware to perform real-time sensing of GMA welding. Electrical, optical, thermal, acoustic, and multimodal systems can be identified as the dominant solutions in terms of welding process state monitoring and defect detection [5].

Electrical monitoring is the most mature and commonly used technique because current and voltage are mostly directly available from the welding power source and reflect arc length, short-circuit events, and transfer regularity. Early adoption of electrical parameter monitoring was based on statistical and signal processing methods using average current, voltage, and short-circuit frequency. Processing and analysis results were used to detect process deviations. Nowadays, modern systems use neural networks or other machine learning models to associate features of electrical signals waveforms with defects like porosity, lack of penetration, or incorrect bead geometry [5,6,7,8]. For example, voltage-signal features extracted in short time intervals have been used with deep neural networks to detect and predict porosity in GMA welding without additional inspection hardware [6], and empirical mode decomposition features combined with extreme learning machines have been used for GMA weld quality diagnosis [8]. However, electrical signals are not always sufficient as diagnostic signals. They can indicate arc stability, short-circuiting, droplet transfer, or heat input changes. However, they are indirect signals: they do not directly reflect weld pool shape, penetration depth, bead geometry, porosity, lack of fusion, or internal cracks.

Vision systems with observation devices running in various modalities and wavelengths have consequently become an important part of modern arc welding monitoring. Laser vision sensors, high-speed cameras, narrow-band optical filtering, and structured-light systems are useful when surface features, like bead profile, weld pool geometry, and seam position, need to be investigated. Additionally, despite intense arc radiation, fumes and spatter metal transfer type and quality can be assessed when the properties of the cameras and illuminators are properly selected [9,10,11,12,13,14,15]. The application of multidimensional datasets (like sequences of video frames) is driven by the rapid development of deep learning algorithms and hardware that supports machine learning. Convolutional neural networks (CNNs) have been applied to analyze images online to detect defects in robotic arc welding [10], image based analysis has been used to characterize GMA metal transfer [11], and weld pool image sequences have been processed with CNN-LSTM (Long Short-Term Memory) architectures to estimate penetration states in real time [12,15]. All obtained results and industrial applications show the power and future potential of vision-based monitoring systems. There are also severe disadvantages that hinder weld monitoring using machine vision: practical deployment can be constrained by line-of-sight requirements, welding arc glare, smoke, lens contamination, camera positioning, and the need for optical access near the hot welding torch. Moreover, for cheaper devices, the spatial resolution, as well as acquisition frequency, cannot sufficiently capture the dynamic and rapidly changing phenomena in the welding area and in the created seam.

Acoustic, ultrasonic, and vibration-based methods provide a complementary route because similar phenomena generate arc sound, acoustic emission, and structural vibration. In GMA welding, especially when the torch is fixture-mounted, the main sources of vibration (of different frequencies) are wire feed mechanism vibration, arc force, and metal transfer pulsations. In the first case, when the wire is physically passing through the torch, all disturbances in wire feeding caused by drive rolls, wire spool, linear friction, and small stick-slip variations can excite the torch for abnormal vibrations. In the second case, droplets that form, detach, and transfer into the weld pool repeatedly create periodic force disturbances in the welding torch assembly. As a result, these disturbances appear as wire and torch shaking. Optical–acoustic fusion has been used for short-circuiting gas metal arc welding (GMAW-S) quality assessment [16]. Air-coupled acoustic emission and weld-input features have supported machine learning prediction of GMA weld quality [17], and MAG (Metal Active Gas) experiments have shown that airborne and contact acoustic emission signatures can reveal imperfections and support decisions on whether fabrication should be halted before completion [18]. The non-contact principle of the measurement of sound and structural acoustic emission [19] ensures a rapid response for defect creation when monitoring welding. These results support the hypothesis that the mechanical response of torch–workpiece systems, emerging in the form of vibrations, can convey certain diagnostic information about process stability and joint quality. Moreover, this information is at least partially independent from electric signals. Infrared thermography and fused infrared–vision imaging have also been used for welding process and heat-affected-zone assessment [20,21].

The state of the art has shifted in recent years from single-parameter monitoring with a fixed threshold toward machine learning solutions that combine supervised or unsupervised models trained over signals of different dimensions and modalities. Combinations consist of high-frequency signals, short time windows, frequency features, or time–frequency features of acquired measurement signals. In wire arc additive manufacturing and related GMA-based processes, unsupervised learning has been used for online anomaly detection from current and voltage data [22], supervised and unsupervised approaches have been benchmarked for real-time anomaly detection [23], and broader welding reviews stress that industrial adoption requires models that are generalizable, robust to process drift, and deployable with limited labeled data [24]. Supervised deep learning has also been used for back-bead monitoring in GMA welding [25], while CNN-LSTM models have exploited dynamic weld pool image sequences to monitor penetration [15]. More recent online GMA welding studies indicate that acoustic monitoring of transfer mode changes can support real-time defect detection and penetration estimation [26].

Despite enormous progress in monitoring, a gap remains between in-process defect and inconsistency detection and operationally useful early recognition of process state’s influence on joint quality. As time window or frame-level accuracy is reported, a final bead classification is still the most popular and reliable approach. Moreover, even fewer reports deal with the task of quantifying how much of the seam must be observed before the final quality state becomes separable. This distinction is critical for process control. A model that identifies which weld is unacceptable after the whole joint was made is useful for inspection and post-process quality control. A model that recognizes the suspected state within the first few millimeters after the defect has formed may enable corrective parameter adjustment, as well as fast, safe, and controlled process termination that allows for a rework of already produced joints or upgrading of the manufacturing technology before design errors or process implementation errors propagate to subsequent joints.

This paper addresses the following question: how early during the pass can the final quality state be recognized from the already observed part of the seam? The aims of this study, therefore, were as follows: (i) to determine the minimum observed seam length from which the final quality state of a GMA weld becomes statistically separable, using a laser Doppler vibrometer as the only diagnostic sensor; (ii) to convert this prefix-based verdict into a sequential online alarm and to quantify the lead time it provides before the pass ends; and (iii) to establish how the resulting decision earliness depends on the welding parameters, in particular travel speed. This is deliberately framed as early recognition of the final weld state rather than as anticipation of a mid-weld defect onset, because the defective welds in the present dataset are defective along their full length. The main contributions of this paper are as follows:

- A causal prefix prediction approach, which estimates the final weld condition based only on the already observed initial part of the seam and presents an earliness curve with bootstrap confidence intervals.
- A fully streaming version of the method, which updates the probability of a defective weld approximately every 50 ms using only causal cumulative statistics.
- A sequential online decision rule that converts the probability stream into alarms with a measurable recognition lead relative to weld completion and facilitates characterization of its false-alarm behavior under the constraint of a small acceptable-weld set.
- A speed-stratified analysis of decision latency, showing that latency is approximately constant in time so that spatial decision position and remaining lead scale with welding speed (associations with voltage and wire feed are reported descriptively, since these parameters covaried by design and cannot be isolated).
- A critical discussion of dataset-related limitations, including class imbalance, whole-weld labels, and the difference between early recognition and anticipation of defect onset.

## 2. Materials and Methods

### 2.1. Specimens, Welding Setup, and Data Acquisition

**Figure 1:**
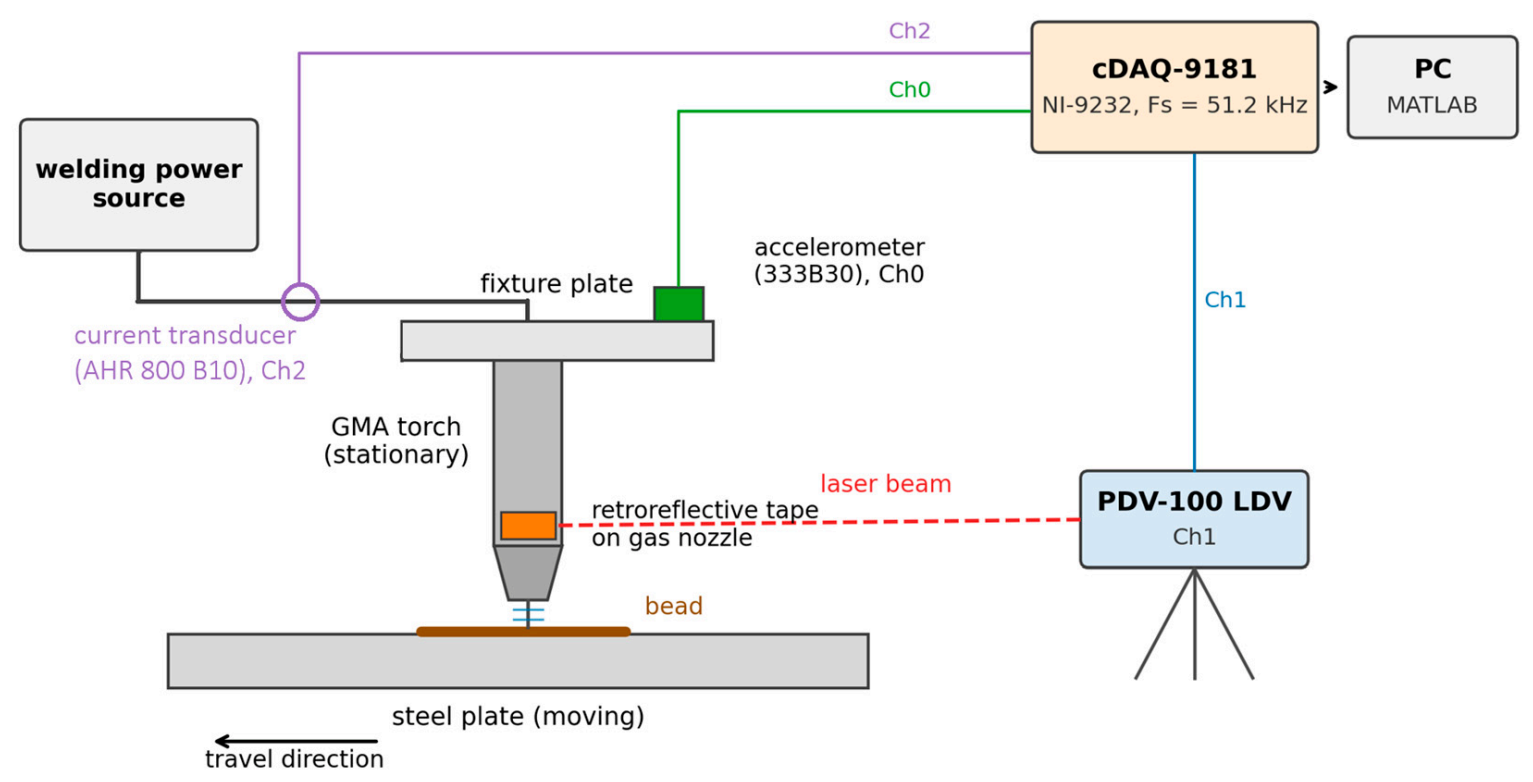
Schematic of the experimental setup. PDV-100 denotes the portable laser Doppler vibrometer measuring torch vibration velocity, and cDAQ denotes the NI cDAQ-9181 data acquisition chassis with the NI-9232 module that digitized all three channels at 51.2 kHz.

The experiment monitored a GMA welding process under stable and deliberately destabilized conditions. Bead-on-plate deposits were produced using an ESAB Warrior Edge 500 CX (ESAB Corporation, North Bethesda, MD, USA) power source and a manually set GMA program. The consumable electrode was ESAB (ESAB VAMBERK, s.r.o., Vamberk, Czech Republic) AristoRod 12.50, diameter 1 mm (EN ISO 14341-A: G 3Si1 [27]), with EN ISO 14175: M21 [28] shielding gas (Ar + 18% CO_2_) supplied at 11.5 L/min through a 12 mm nozzle on a liquid-cooled torch. The torch was held normal to the plate with a 20 mm electrode stick-out, while the plate moved beneath the stationary torch. Bead-on-plate deposition was selected deliberately because it isolates the arc and metal transfer dynamics from the geometric effects of a prepared joint. The vibration signature exploited in this work originates in the arc force, short-circuit ruptures, and droplet detachment, and these phenomena are governed by the same electrical and thermal mechanisms in bead-on-plate and in grooved or fillet joints. What bead-on-plate deposition does not reproduce is the influence of joint geometry itself, namely root gap variation, sidewall heat extraction, restraint, and multi-pass thermal history, all of which modify the weld pool and may add their own low-frequency components to the measured signal. The configuration is therefore representative for the process-stability classification addressed here, but the transferability of the trained model to real joint geometries has to be re-verified experimentally, and this is stated as a limitation in Section 4. A schematic representation of the test stand is presented in Figure 1. The parameter sets are listed in Table 1; the voltage and wire feed values given there are the values adjusted on the power source, whereas the welding current results from the self-regulation of the process and was measured rather than set. The measured current also quantifies the destabilization imposed by the protocol. The within-pass standard deviation of the current stays between 3.62 and 5.19 A for every set welded at 18 V and 21 V and rises to 16.31–28.50 A for the sets welded at 12 V and 16 V. The sets destabilized by excessive travel speed alone (T8–T10 and T23–T25) therefore keep a current variability indistinguishable from that of the acceptable sets, which shows that the deviation imposed in those trials is one of bead geometry rather than of arc stability.

**Figure 2:**
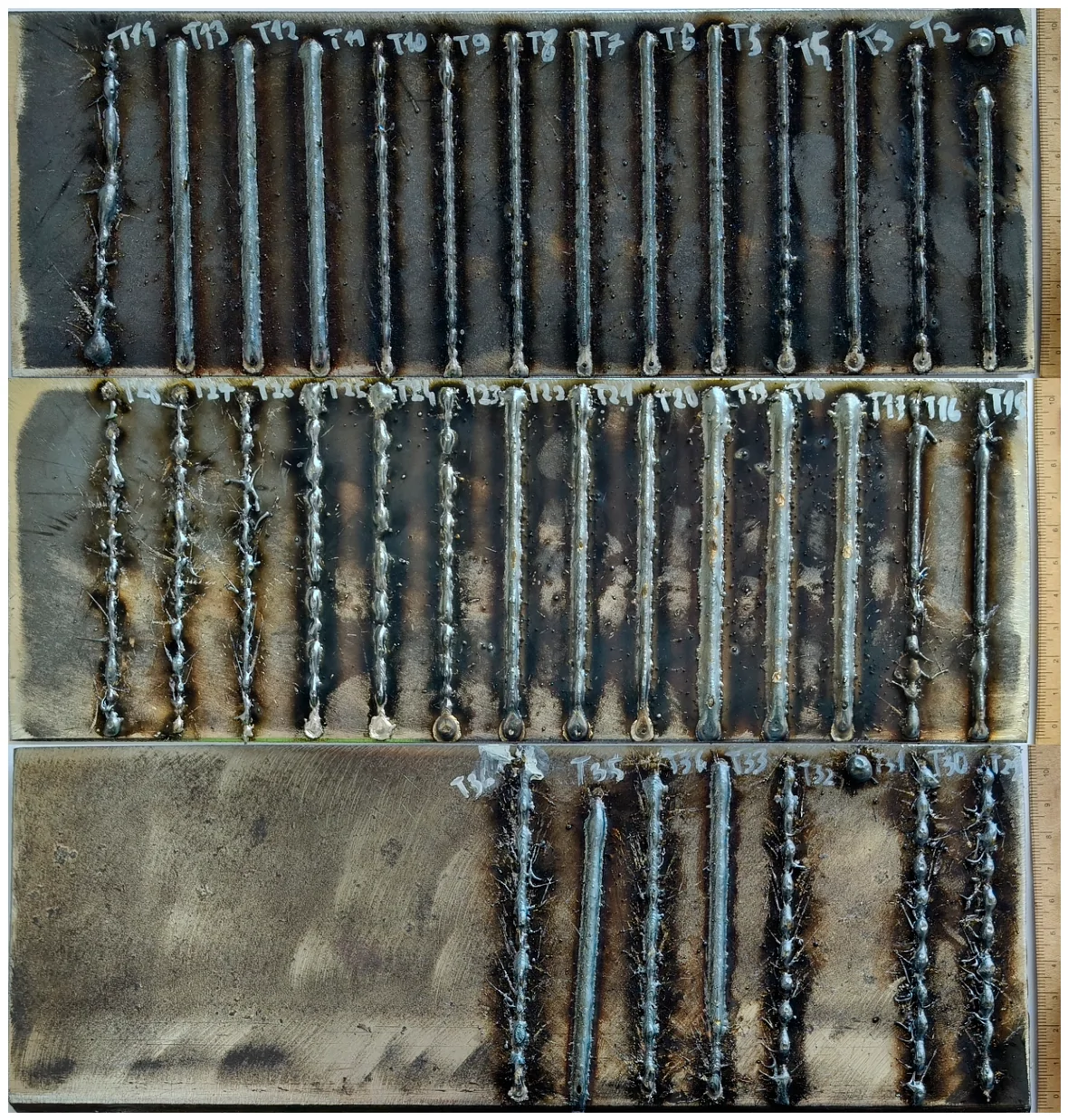
Representative bead-on-plate specimens produced in the trials: stable (**top**), moderately destabilized (**middle**), and strongly destabilized (**bottom**) welding conditions.

**Figure 3:**
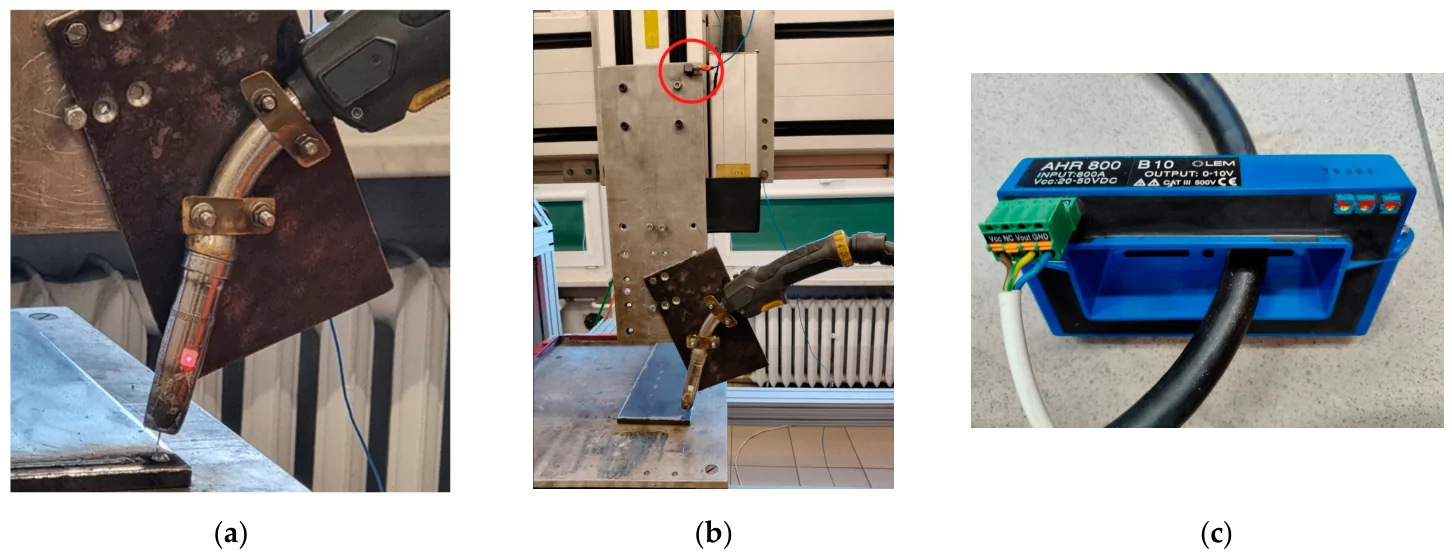
Measurement instrumentation used in the experiments: (**a**) PDV-100 portable laser Doppler vibrometer (Channel 1, torch vibration velocity); (**b**) 333B30 piezoelectric accelerometer (marked with red circle) mounted on the fixture plate (Channel 0); (**c**) AHR 800 B10 split-core Hall-effect current transducer (Channel 2, reference welding current).

The dataset contains 32 GMA clads (Figure 2). Trials T1–T4 were required to calibrate the measurement setup and welding device. Each parameter set in Table 1 was repeated in three (in two cases, four) nominally identical passes, so the dataset contains replicated welds for every condition rather than a single weld per parameter set; the designators in Table 1 identify the repetitions belonging to each set. The repetitions were made consecutively on separate plates with the torch and the vibrometer left undisturbed, so they capture the run-to-run variability of the process itself, which is the variability that an early-recognition system has to tolerate. Replication at this level was chosen in preference to a larger number of distinct parameter sets because the leave-one-weld-out protocol requires several independent welds per condition in order to give a meaningful estimate of between-weld variability. Weld labels denote the process-stability class imposed by the experimental protocol (stable versus deliberately destabilized), together with expert visual assessment of the resulting bead; they were not independently confirmed by radiographic or metallographic examination. Accordingly, the classification task separates stable from destabilized welding conditions rather than verified metallurgical defect types. The nominal bead length was approximately 100 mm, confirmed by measurement; two specimens (T31 and T35) were shorter, as discussed in Section 3.1. Three time-synchronized channels were acquired at Fs = 51.2 kHz with a cDAQ-9181 data acquisition chassis fitted with an NI-9232 sound-and-vibration module (National Instruments, Austin, TX, USA) and streamed to a PC running MATLAB 2026a (MathWorks, Natick, MA, USA). Channel 0 recorded the general mechanical vibration of the fixture, in the form of acceleration, with a 333B30 piezoelectric accelerometer (PCB Piezotronics, Depew, NY, USA; sensitivity 100 mV/g, frequency range 0.5–3000 Hz, Figure 3b) attached with double-sided adhesive tape to the steel plate to which the welding torch was clamped. Channel 1 recorded the vibration velocity of the torch with a PDV-100 portable laser Doppler vibrometer (Polytec GmbH, Waldbronn, Germany; He-Ne laser, frequency range 0–22 kHz, Figure 3a), mounted on a tripod placed at a distance of 2 m from the arc with the laser spot aimed at a strip of retroreflective tape on the gas nozzle, providing a non-contact measurement directly at the torch. Channel 2 recorded the welding current with an AHR 800 B10 split-core Hall-effect current transducer (LEM International SA, Meyrin, Switzerland; nominal current 800 A, peak current 2500 A, 0–10 V output, Figure 3c) clamped around the power cable feeding the torch. The LDV signal was the only diagnostic channel used for classification. Current was used only for offline arc segmentation and as an independent reference, and the accelerometer was retained only as an auxiliary reference. Each measurement also contained operator-triggered noise before and after the pass. All three measuring channels were calibrated before the experimental campaign. The PDV-100 vibrometer was used with the manufacturer’s factory calibration, valid at the time of the trials, and its velocity output was verified against a reference shaker excitation. The 333B30 accelerometer was calibrated with a handheld reference exciter at 159.2 Hz, and the Hall-effect current transducer was calibrated against a reference clamp meter over the working range of the power source.

### 2.2. Arc Segmentation

The region of effective welding was automatically isolated from the reference welding current channel. This step was necessary because, in each welding pass, the start of measurement was not synchronized with the start of welding arc glowing. To achieve this, the envelope of the current signal was examined, and it was near zero before and after the welding (when the arc was glowing). A robust threshold was set between the noise floor, estimated from the first 0.1 s of the recording, and the arc level, estimated by the 90th percentile. Short drops in the middle of the arc, shorter than 0.30 s, were filled using morphological gap filling. Next, longest continuous segment where arc was detected was selected if its length was at least 0.15 s. Both ignition and extinction parts were trimmed using a 0.15 s interval. In this manner, the whole arc duration was retained. Using the welding speed described in Section 2.1, mapping between arc-on time and bead length was achieved. Therefore, the position along the bead was calculated as the window time multiplied by the welding speed. This mapping assumes that the carriage travels at the nominal speed for the whole arc-on interval. It therefore overestimates the deposited length whenever current flows without corresponding travel or deposition, for example, when the electrode stubs against the plate or when current persists after the carriage has stopped; specimen T31 is the one case in this dataset where the effect is large.

### 2.3. Feature Calculation

Feature extraction is an important step in early in-process detection of welding defects because it transforms raw sensor signals into descriptive parameters that can be analyzed before the weld is completed. Properly selected features make it possible to identify early changes in process behaviors, reduce the complexity of the data, and support faster detection of conditions that may lead to defective welds [29,30].

Before feature calculation, the signals were band-pass-filtered using fourth-order filters. The frequency ranges were 20–22,000 Hz for the LDV signal and 20–3000 Hz for the accelerometer signal. In addition, narrow notch filters with Q = 35 were applied at 50 Hz and its harmonics up to the fifth order. It was introduced to the processing path in order to reduce interference from the power supply network. The lower cut-off frequency of 20 Hz was used to remove quasi-static mounting plate motion and low-frequency structural vibrations of the fixture, which do not contain useful arc-related information. At the same time, the wide upper frequency range of the LDV channel was retained to preserve broadband components generated by short-circuit ruptures, consecutive arc ignitions, and finally droplet detachment.

After preliminary studies, we chose to calculate 38 features in 0.10 s windows with 50% overlap. The selected window length was a compromise between two factors. Firstly, this was the minimal acceptable window length, because the window should be as short as possible to treat the process as quasi-stationary and to provide a fast update rate for the streaming scenario. On the other hand, the time window should be long enough to include several short-circuit cycles and to ensure a spectral resolution of 10 Hz. Because trials were performed with different welding speeds, one window that has constant realization time covers approximately 0.8–6.7 mm of seam. Therefore, the spatial resolution was mainly determined by the welding speed. The 50% overlap was intentionally selected to reduce the risk of possible short transient event division between two neighboring windows. All features used in the investigations were gathered in four complementary groups (Table 2). The four groups in Table 2 are complementary in what they describe. The twelve time domain features characterize the amplitude and the impulsiveness of the torch vibration, and therefore, they respond to the rate and the violence of short-circuit ruptures. The fifteen frequency domain features describe the shape of the power spectrum and the distribution of energy between the six sub-bands, which shifts when the transfer mode changes. The three envelope features quantify the amplitude modulation of the signal, whose dominant frequency corresponds to the short-circuit repetition rate. The eight wavelet-packet energies resolve transient, non-stationary events that are smeared out by the stationary spectral estimators. Each of the 38 features is computed independently for every 0.10 s window, so one window is described by a 38-dimensional vector.

The first group consists of the following time domain features: RMS, peak, peak-to-peak, standard deviation, skewness, kurtosis, crest, shape, impulse and clearance factors, zero-crossing rate, and energy. This set consists of well-established statistical descriptors that are commonly used in vibration-based condition monitoring [29]. These features reflect different behavioral aspects of vibrational signals. RMS and energy track the overall excitation level of the torch–workpiece system. Kurtosis, crest, impulse, and clearance factors are sensitive to impulsive events such as short-circuit ruptures, spatter impacts, and irregular ignitions of arc occurring during one seam creation. All of those phenomena become more frequent and more random when the process destabilizes.

Second, the most abundant group contains frequency domain features: spectral centroid, spread, skewness, kurtosis, entropy, roll-off, dominant frequency, flatness, total power, and six normalized band-power ratios computed in the bands 20–200, 200–1000, 1000–3000, 3000–6000, 6000–11,000, and 11,000–22,000 Hz. These shape descriptors provide a compact and low-dimensional description of the power spectrum [30]. To ensure comparability of feature values, band power ratios were normalized by the total band power. This resulted in redistribution of vibration energy between frequency sub-bands. Thus, there were no changes in band absolute power level. The applied normalization also makes these ratios more reliable and less sensitive to changes in LDV observation distance and general excitation level.

The next group of features, gathering envelope-analysis-based ones, is connected to the time domain group. In this case, features are computed from the analytic signal obtained by the Hilbert transform: envelope RMS, envelope kurtosis, and modulation frequency. Envelope analysis is a classical tool that allows for detection of impulses in signals with modulated amplitude. It is commonly used in machinery diagnostics [31], especially when dealing with rolling bearing fault diagnostics. In the context of the performed research, the dominant modulation frequency was expected to be correlated to short-circuit repetition rate and weld pool oscillations. Both are direct markers of welding arc stability and joint quality.

The last group of considered features is the result of wavelet decomposition. The energies of the eight terminal sub-bands of a level-3 MODWPT decomposition were calculated to reflect non-stationary and transient fault signatures. This approach is widely applied for various mechanical systems [32,33].

No separate feature selection stage was applied. The complete 38-dimensional feature vector was used as the input to the random forest models. These models perform an implicit feature ranking through impurity-based split selection. This approach keeps the processing pipeline simple and avoids an additional feature selection step, which could introduce information leakage between welds in the leave-one-weld-out validation protocol. Each window was assigned its center time and position along the bead. For the supervised analysis, each window inherited the label of its parent weld.

### 2.4. Detection and Early Prediction Methodology

**Figure 4:**
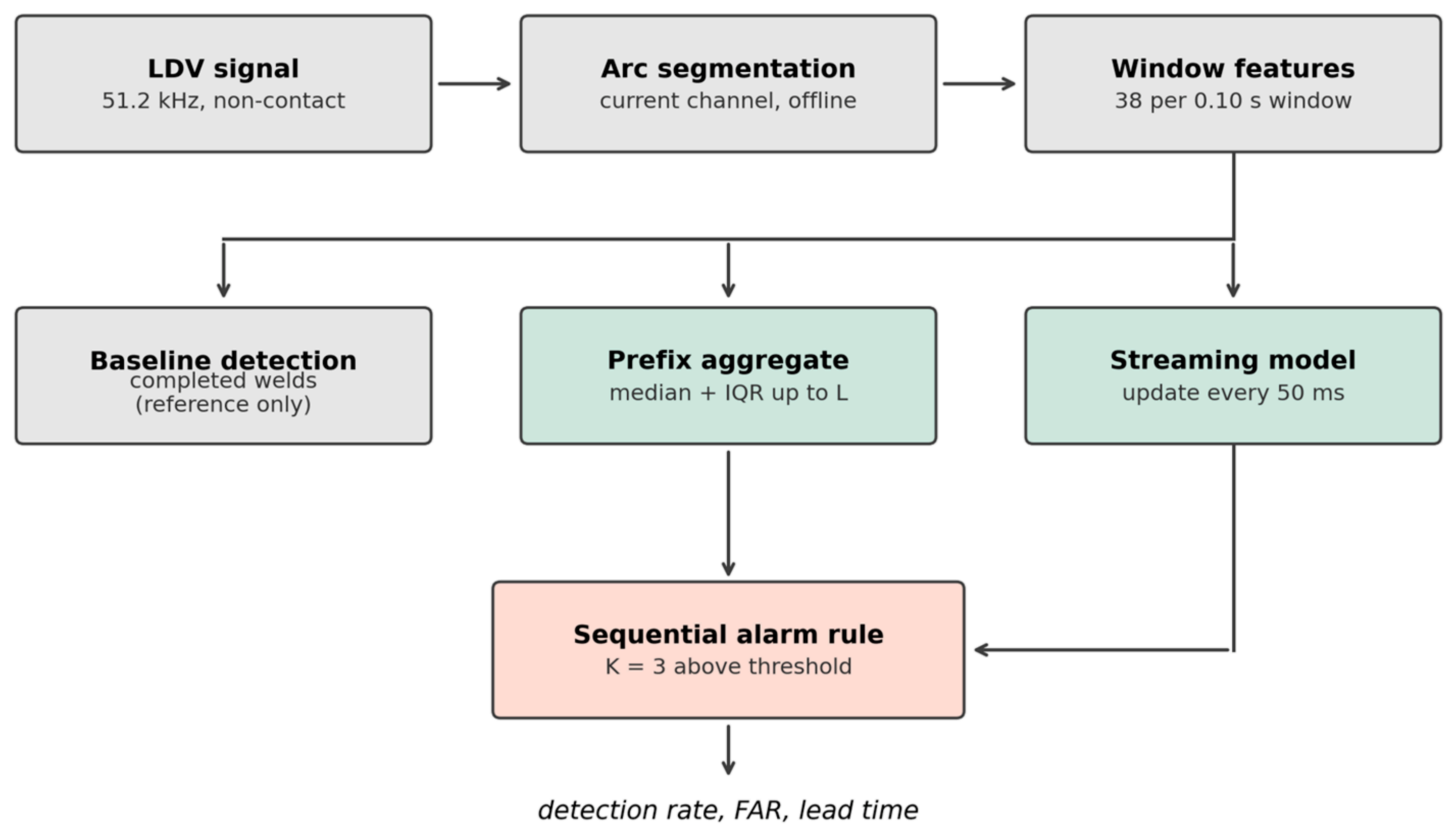
Overall processing pipeline.

Before introducing and proposing a solution for the early prediction task, a random forest classifier was trained on the time window level. It was made with the already mentioned leave-one-weld-out groups and then aggregated to make decisions on the whole weld level. The overall processing pipeline, from the raw LDV signal to the online alarm, is presented in Figure 4.

The baseline detectors fall into three groups. The first group included statistical monitors. These methods operated on the standardized window features and included the PCA Hotelling’s T^2^ statistic, the PCA Q statistic (also known as the squared-prediction-error statistic), and the Mahalanobis distance to the acceptable-class model with regularized covariance. Hotelling’s T^2^ statistic is able to describe the variation inside the retained principal component subspace, where the number of components was selected to reach the prescribed cumulative variance. Next, the PCA Q statistic described the residual variation outside the considered subspace. As all statistics were calculated on the window level, to obtain one value for each weld, simple averaging was applied.

The second group included signal descriptors calculated directly from the raw LDV waveform of each weld. These descriptors included the spectral distance, defined as the Jensen–Shannon divergence between the normalized Welch spectrum of a given weld and the median spectrum of welds pointed by expert as correct ones. They also included the maximum spectral kurtosis, used as an indicator of transient components. Next was the peak of the Hilbert-envelope spectrum in the 2–500 Hz modulation band normalized by its mean level. Finally inverse LDV–accelerometer coherence was also calculated (low coherence can be understood as a beginning of abnormal vibration signature).

The third group included machine learning detectors. These methods used features aggregated to the weld level. Five one-class models were trained only on acceptable welds: a one-class SVM with an RBF kernel, an isolation forest, a local outlier factor model, the negative log-likelihood of a Gaussian mixture fitted in the space of the first principal components, and an autoencoder reconstruction error. An additional two supervised classifiers were used: a random forest classifier and an SVM.

To increase the credibility of the obtained results, a fused detector was used. It was designed to use detectors with AUC scores higher than 0.6 and aggregate their outputs using a robust median/MAD (Median Absolute Deviation) combination.

For quantifying the earliness, the features were aggregated only from windows with a position that was less than or equal to an observed initial length L. The aggregate consisted of the median and the interquartile range of the standardized window features, which gives a 76-dimensional prefix representation. This combination was chosen because it is robust location–dispersion summary and is not sensitive to isolated outlier windows. A random forest (100 trees per fold; for the unknown welds, a 200-tree model retrained on all labeled welds was used) was trained and evaluated with leave-one-weld-out grouping, while L was moved from 5 to 100 mm with a 5 mm step. The obtained earliness curve reports AUC, accuracy, sensitivity, and specificity as functions of the observed seam length. The 95% confidence intervals were obtained by bootstrapping of welds (1000 replicates) using the out-of-fold scores. The minimum lengths for reaching AUC ≥ 0.90 and AUC ≥ 0.95 were recorded.

The aggregate scheme fixes the length L, but a deployment system should update its verdict continuously. Therefore, in the streaming variant, for each window k of a weld, a causal cumulative representation was formed. The running mean and running standard deviation of the standardized features over windows 1…k were concatenated together with the current window (114 features per one update). The classifier sees both the accumulated history of the weld and its instantaneous state. The causality is then well preserved because no future window enters into the representation. A single position-agnostic random forest (120 trees per leave-one-weld-out fold) was trained on the pooled cumulative features of all training welds and applied on the held-out weld. This resulted in trajectory P(defective), which was updated approximately every 50 ms. The streaming earliness curve was evaluated at each length L by taking the value of this trajectory at the last window not exceeding L.

**Figure 5:**
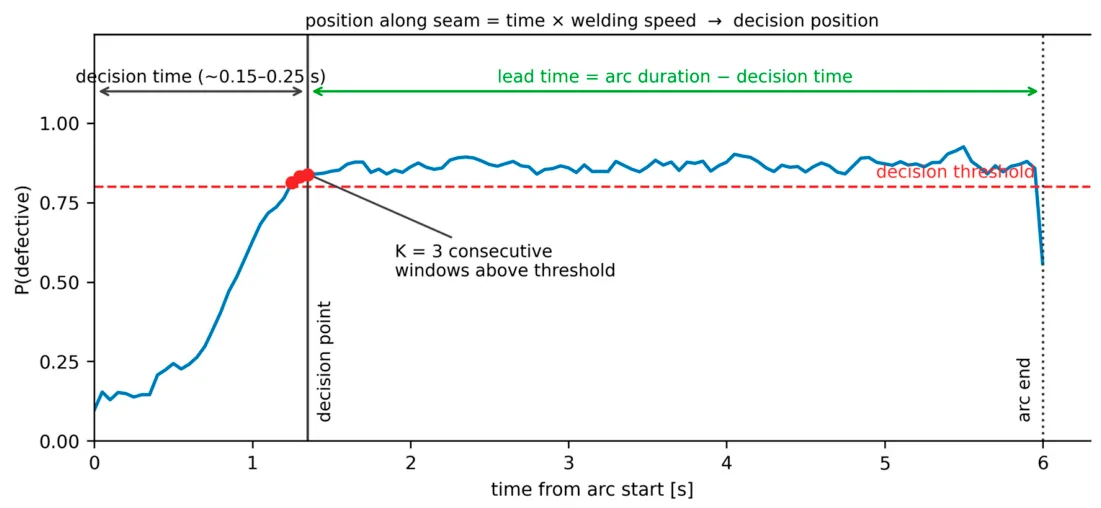
Illustration of the online decision quantities: the alarm is raised after K = 3 consecutive smoothed values of P(defective) (blue line) exceed the decision threshold. The decision time is nearly constant in time, while the decision position and the remaining lead time scale with the welding speed.

Finally, to obtain the sequential online decision rule, the probability stream was smoothed using a causal trailing moving average over the last three windows. The alarm was raised after K = 3 consecutive smoothed values exceeded the decision threshold. The threshold varied from 0.20 to 0.80 with step 0.05 (up to 0.95 in case of the streaming variant). The persistence length K and the smoothing window were both set to three consecutive windows on the basis of the window geometry, rather than being tuned on the test data. With 0.10 s windows and 50% overlap, one window update corresponds to 50 ms, so three consecutive confirmations correspond to approximately 0.20 s of arc, which is the shortest interval that spans several short-circuit cycles at the transfer frequencies observed here and is therefore long enough to reject an isolated single-window excursion caused by one spatter impact. Values K = 2 and K = 4 were checked—K = 2 produced additional false alarms on acceptable welds, whereas K = 4 delayed the decision by a further 50 ms without reducing the false-alarm rate. The decision threshold was not fixed a priori but swept over the range given above, and the reported operating point was selected from that sweep by the false-alarm requirement (Section 3.5). For each weld, this gave the decision position, decision time, and lead time, defined as the arc duration minus the decision time. For acceptable welds, any alarm was counted as a false alarm. By sweeping the threshold, the trade-off between detection rate, false-alarm rate (FAR), and lead time was produced. The definitions of the decision time, decision position, and lead time are illustrated in Figure 5.

All supervised evaluations used leave-one-weld-out grouping to prevent window-level leakage. AUC was computed by the Mann–Whitney statistic. Operating thresholds were selected by maximizing Youden’s J using out-of-fold scores, and confidence intervals used weld-level bootstrap resampling. Dependence of decision earliness on process parameters was assessed by Spearman rank correlation. Feature standardization used a variance floor equal to 1% of the median non-zero standard deviation estimated on acceptable windows, preventing near-constant features from destabilizing distance-based scores.

## 3. Results

### 3.1. Segmentation Consistency

**Figure 6:**
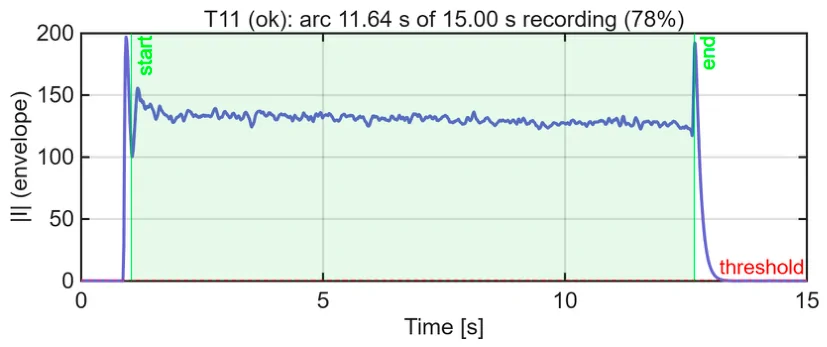
Exemplary segmentation of welding realization, based on current signal.

After segmentation based on the raw welding current signal, the detected arc covered the full part of welding process realization, where the welding arc was glowing. An example segmentation result is in Figure 6. The arc duration differed considerably between various weld speeds. It ranged from approximately 1.5 s at 66.66 mm/s to almost 11.6 s at 8.33 mm/s. Knowing the welding speed, mapping between welding time and joint length could be conducted. Analyzing the obtained results, good correspondence to real seam length was obtained. All clads were about 100 mm long. Two specimens deviated from this length. In T31, the value of approximately 24 mm reported by the segmentation is the arc-on duration converted into traveled distance, that is, the nominal path length corresponding to the interval in which current was flowing, and not the length of the visible deposit. In this trial, the electrode was stubbed against the plate so that relative motion continued without proper metal transfer and current continued to flow after the carriage had already stopped. Both effects add arc-on time that is not accompanied by deposition along the joint, so the time-to-length conversion overestimates the bead length; this is why the specimen appears markedly shorter in the photograph than the segmented arc length suggests. In T35, the arc was extinguished before the end of the intended path, and the deposit was approximately 63 mm long. Both specimens were retained in the analysis, and the limitation of the constant-speed time-to-length mapping is stated in Section 2.2.

### 3.2. Baseline Detection Performance

The supervised window-level model achieved a weld-level AUC of 0.983, while the window-level AUC was 0.931. The model obtained a sensitivity level of 95% (19 of 20 welds with defects or inconsistencies). Only one specimen (T23) was missed. The specificity was 100%, while all six acceptable welds were classified correctly. The ranking of detectors that was created on the basis of the AUC values showed that simultaneously using several one-class models led to AUC = 1.00 for the one-class ensemble. This value should not be over-interpreted—with only six acceptable welds, the negative class is too small to distinguish perfect separation from small-sample coincidence, and the corresponding confidence interval is wide. During the study, we also investigated whether the removal of the initial part of the weld where the arc was ignited and large variations in arc stability can occur will affect the detection performance. It was found that, after analyzing the seam without the first 10 mm, the AUC dropped from 0.983 to 0.967. Therefore, the full seam was retained for the reference detection task.

**Figure 7:**
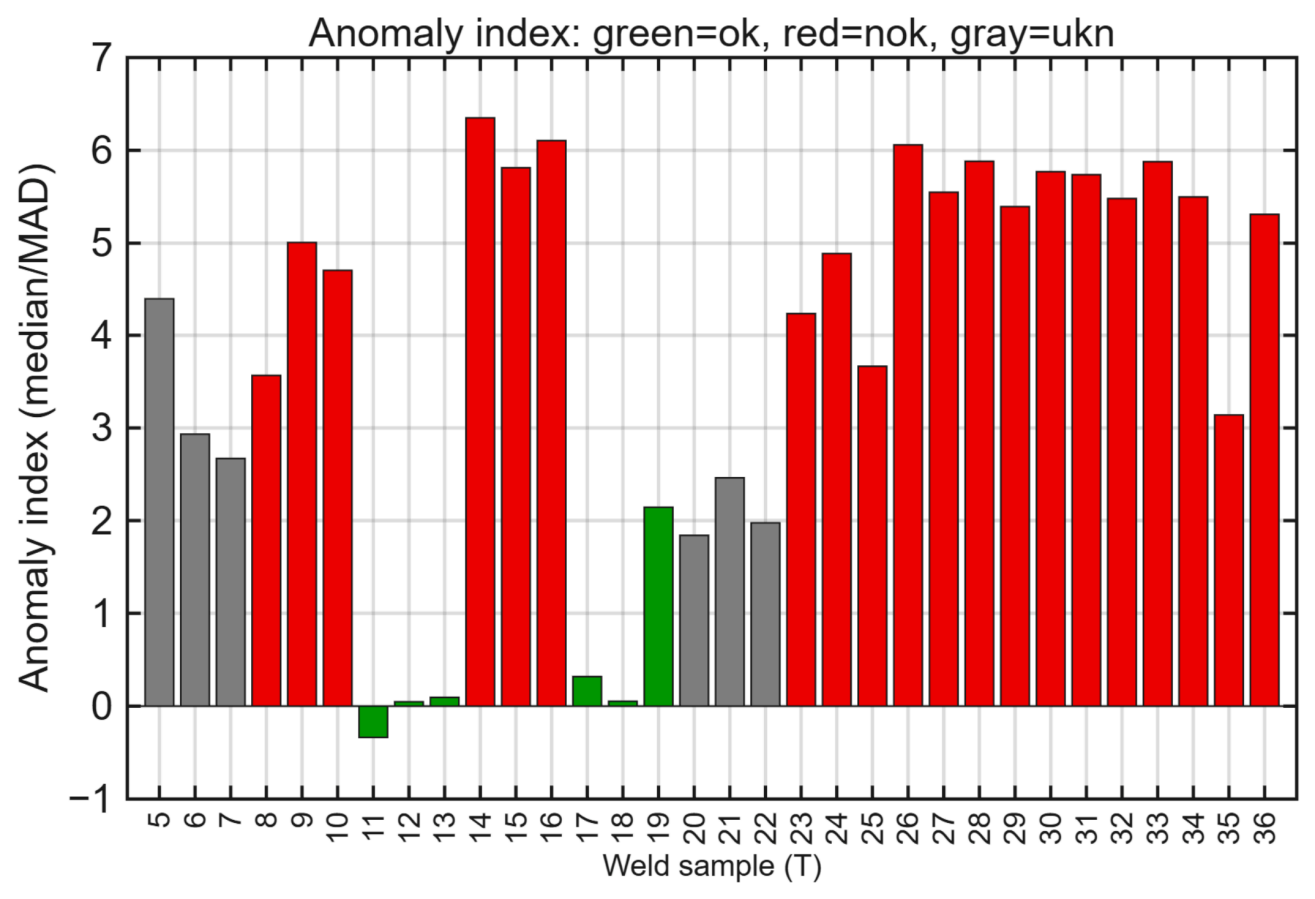
Baseline fused anomaly index per weld.

**Figure 8:**
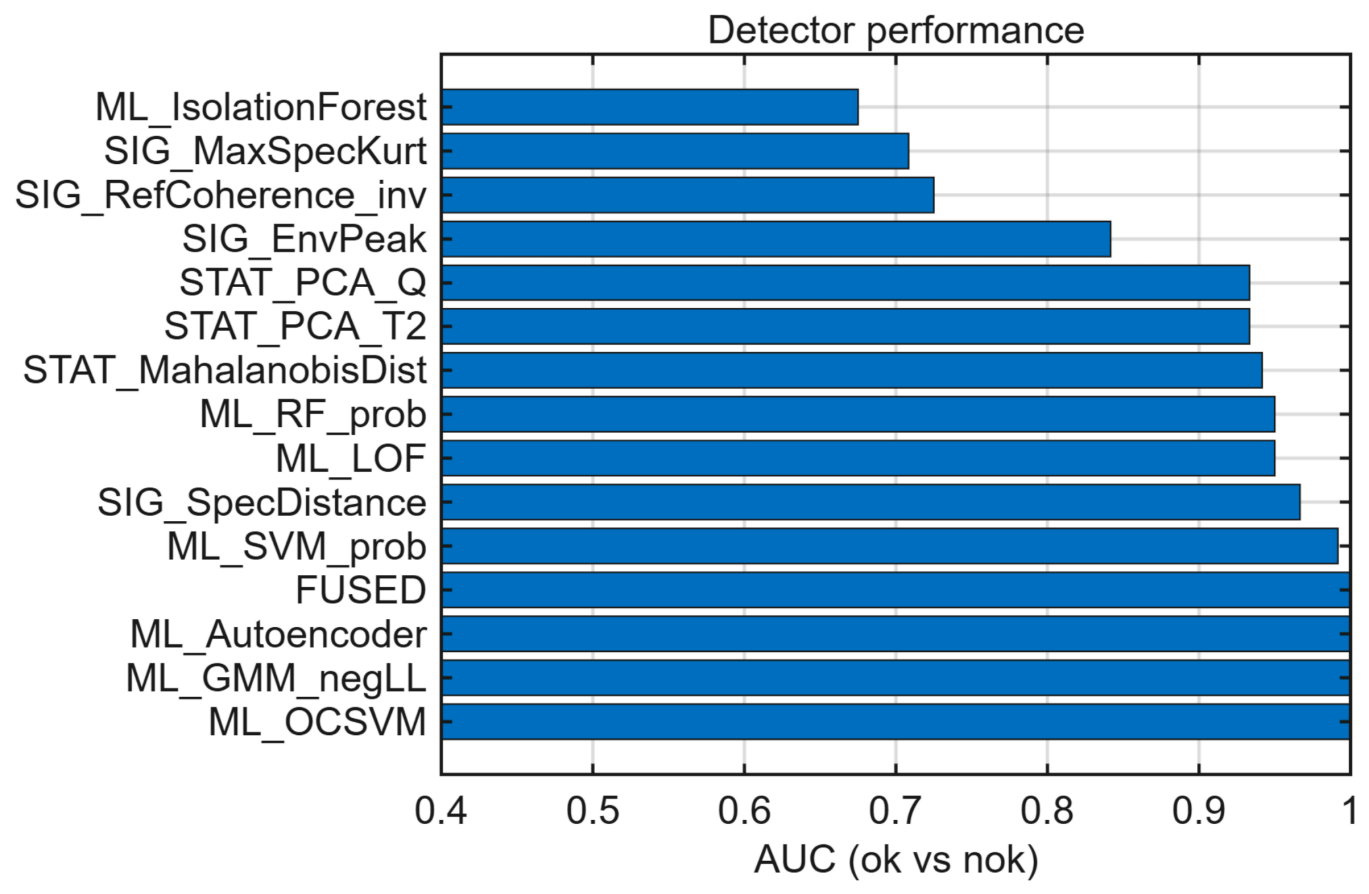
Baseline detector ranking by AUC for acceptable versus defective welds. The vertical axis lists the individual baseline detectors described in Section 2.4 (statistical monitors, raw-signal descriptors, and one-class or supervised machine learning models); the horizontal axis is the weld-level area under the ROC curve obtained for that detector.

**Figure 9:**
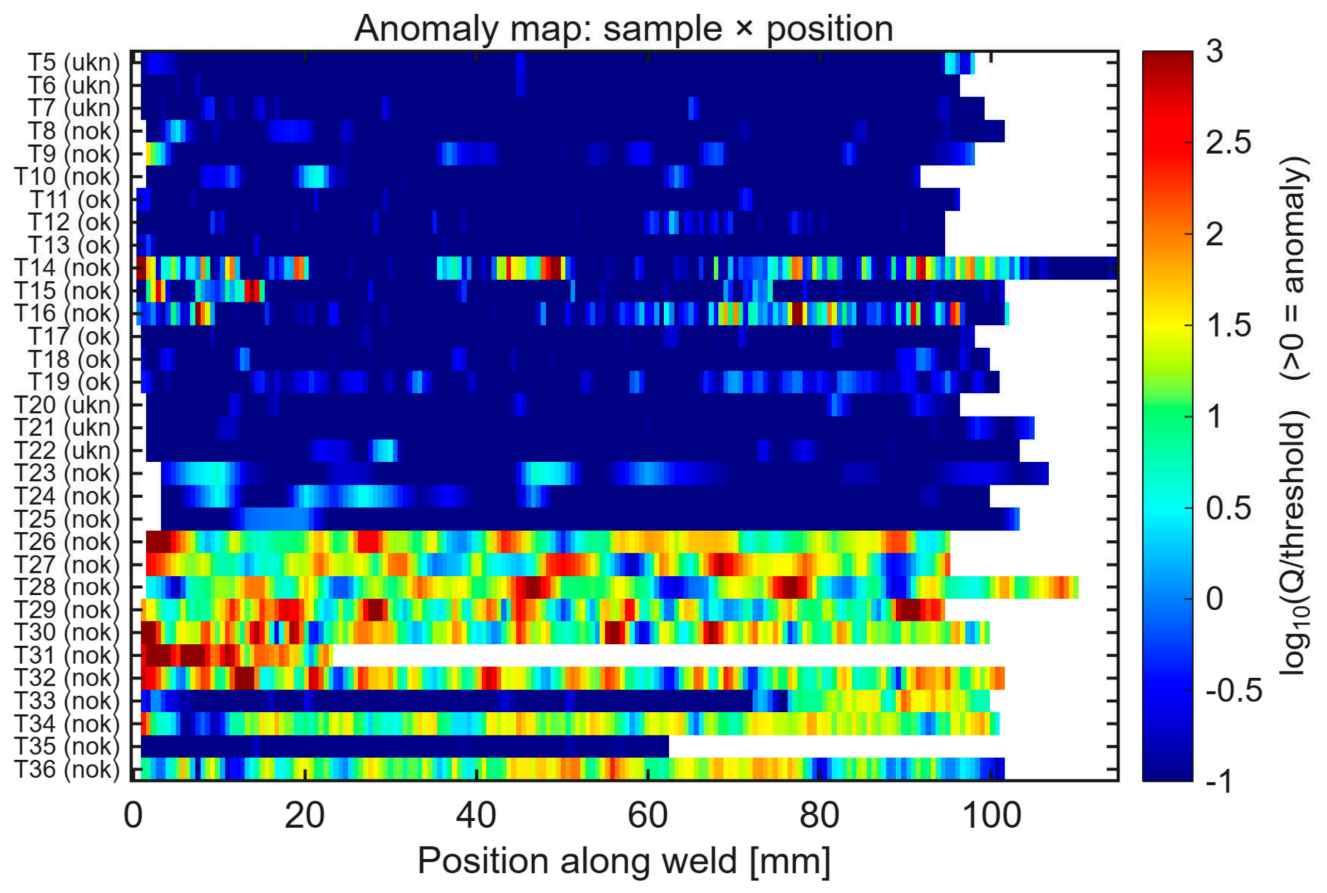
Seam anomaly map. Each row corresponds to one weld, and the horizontal axis corresponds to the position along the bead, with color encoding the local anomaly index. Welds are grouped by label—acceptable (acc), defective (def), and unknown (ukn), with the last denoting the welds whose quality class was not assigned in advance by the experts and those classified by the trained model described in Section 3.9.

These baseline results show that the probability stream derived from the LDV signal is stable and suitable for further analysis, as shown in Figure 7 and Figure 8. The corresponding seam anomaly map is presented in Figure 9. The map was compared with the deposited beads specimen by specimen. The local anomaly index separates the welds according to the character of the imposed destabilization rather than according to their label. In the welds produced at 12 V with a wire feed of 8 m/min, and in two of the welds produced at 16 V, the index is elevated over most of the seam: its median exceeds the acceptable-weld threshold by a factor of 11 to 128, and the longest continuous region above the threshold spans 19 to 54 mm. In the welds destabilized by excessive travel speed, the index behaves as in the acceptable welds. Its median stays between 0.03 and 0.15 of the threshold, against 0.03 to 0.14 for the acceptable welds, and the windows that do exceed the threshold are isolated, with the longest continuous region reaching 3.3 mm against 1.7 mm in the acceptable welds. The welds produced at 12 V with a wire feed of 4 m/min are intermediate. This ordering is only partly reproduced by the weld-level classifier. The classifier recognizes 19 of the 20 defective welds from the same LDV features, and the single weld it misses, T23, belongs to the group destabilized by excessive travel speed, that is to say, the group for which the local index is also uninformative.

**Figure 10:**
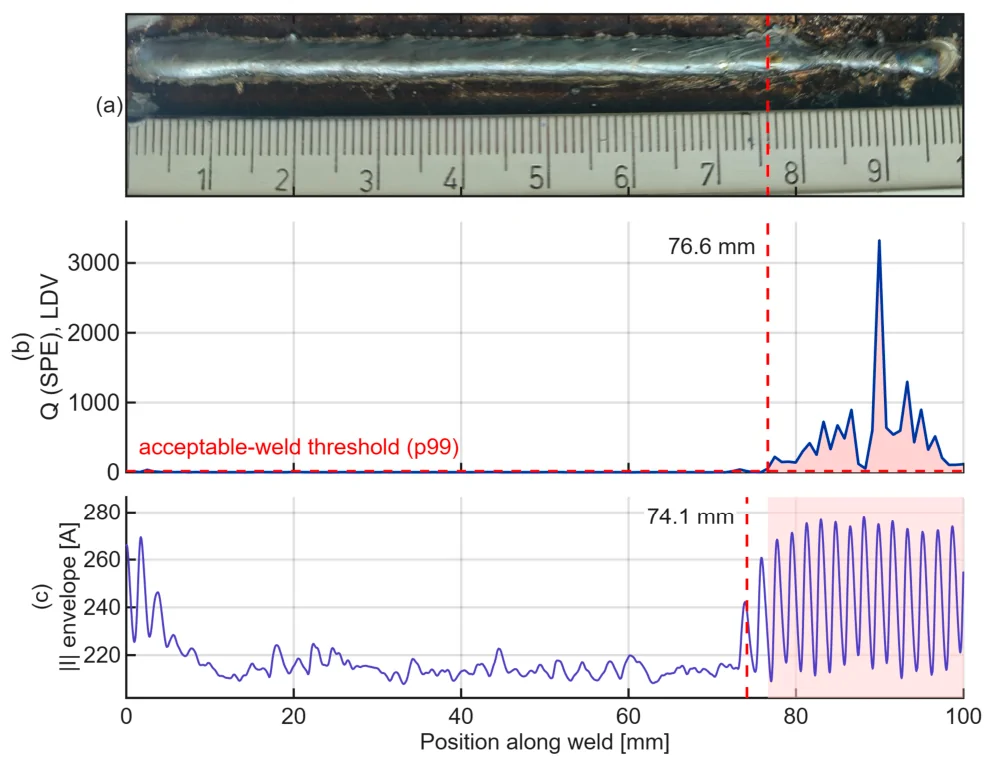
Localized mid-weld transition in specimen T33 (16 V, 8 m/min, 100 cm/min). (**a**) Deposited bead, arc-start end on the left, aligned with the position axis by means of the scale rule; (**b**) local anomaly index Q (SPE) from the LDV channel, with the p99 threshold of the acceptable welds; (**c**) envelope of the welding current, recorded only as an independent reference. The dashed line marks the position at which the anomaly index crosses the threshold.

The two quantities therefore describe different things—the weld-level model exploits the distribution of the window features over the whole pass, whereas the local index marks only those windows whose residual leaves the acceptable-weld subspace, which the irregular short-circuiting at reduced voltage does and the faster but still regular transfer at elevated travel speed does not. One specimen provides a direct check of the localization against the joint itself. In T33, the process is at the baseline level over the first 76 mm; the anomaly index then crosses the p99 threshold of the acceptable welds at 76.6 mm, stays above it in a single continuous region that is 23.3 mm long, and reaches its maximum of 3326 (that is, 176 times the threshold) at 90.0 mm. The median of the same weld remains at 0.07 of the threshold, so this weld is locally defective while appearing acceptable in any statistic aggregated over the whole pass. The welding current, which was recorded only as an independent reference and was never used by the model, shows a comparable transition at 74.1 mm, where the standard deviation of its envelope within a 0.10 s window rises from 1.8 A to 20.8 A (that is, by a factor of 11.5), and the surface of the bead becomes markedly more irregular over the same interval, with the roughness of the intensity profile measured along the bead axis increasing by a factor of about two with respect to the section between 10 and 70 mm (Figure 10). These three observations are mutually independent and agree with each other on the position of the transition, which confirms that the anomaly map localizes a real change in the process state rather than an artifact of the feature representation. A systematic validation against deliberately introduced localized imperfections, nevertheless, remains necessary, since T33 is the only specimen of this kind in the present dataset.

### 3.3. Causal Early Prediction Curve

The earliness curve is reported in Table 3. It is easily noticeable that the final verdict was already separable after the first 5 mm (AUC = 0.94). The minimum observed length reaching AUC ≥ 0.90 was 5 mm, and the minimum length reaching AUC ≥ 0.95 was 15 mm. Discrimination reached AUC = 1.00 over 30–55 mm; the bootstrap interval collapsed to [1.00, 1.00], which reflects the very small number of acceptable welds rather than genuine certainty and should be read as an upper-bounded estimate. The local rapid decrease in results at 10 mm (AUC = 0.85) and the modest decline beyond 75 mm should be interpreted cautiously because of the small number of acceptable welds. A plausible explanation for the performance drop is that windows in this region are still influenced by the transient ignition, which is similar for both classes. However, with 26 labeled welds, sampling variability alone can produce such a fluctuation, as reflected by the wide confidence interval at this length.

The asymmetry between sensitivity and specificity along the curve is also informative. For L, between 15 and 25 mm, the sensitivity already reached 1.00, while the specificity remained at 0.83, meaning that a single acceptable weld was ranked on the wrong side of the operating threshold; with only six acceptable welds, one such weld shifts the specificity by 0.17, so this plateau reflects the granularity of the acceptable class rather than a systematic bias of the model. The slight decrease in AUC beyond approximately 75 mm has a different character. A plausible reading is that, as L grows, the median–IQR prefix aggregate becomes increasingly dominated by the stationary later portion of the seam, so the contrast contributed by the most discriminative early region is progressively diluted, while windows near weld termination add variability of their own. From a deployment perspective, this decline is immaterial, because an early-recognition system would act long before 75 mm of seam is deposited; it does, however, indicate that the prefix aggregate should not be interpreted as a monotonically improving estimator of the final state.

### 3.4. Online Decision and Lead Time

**Figure 11:**
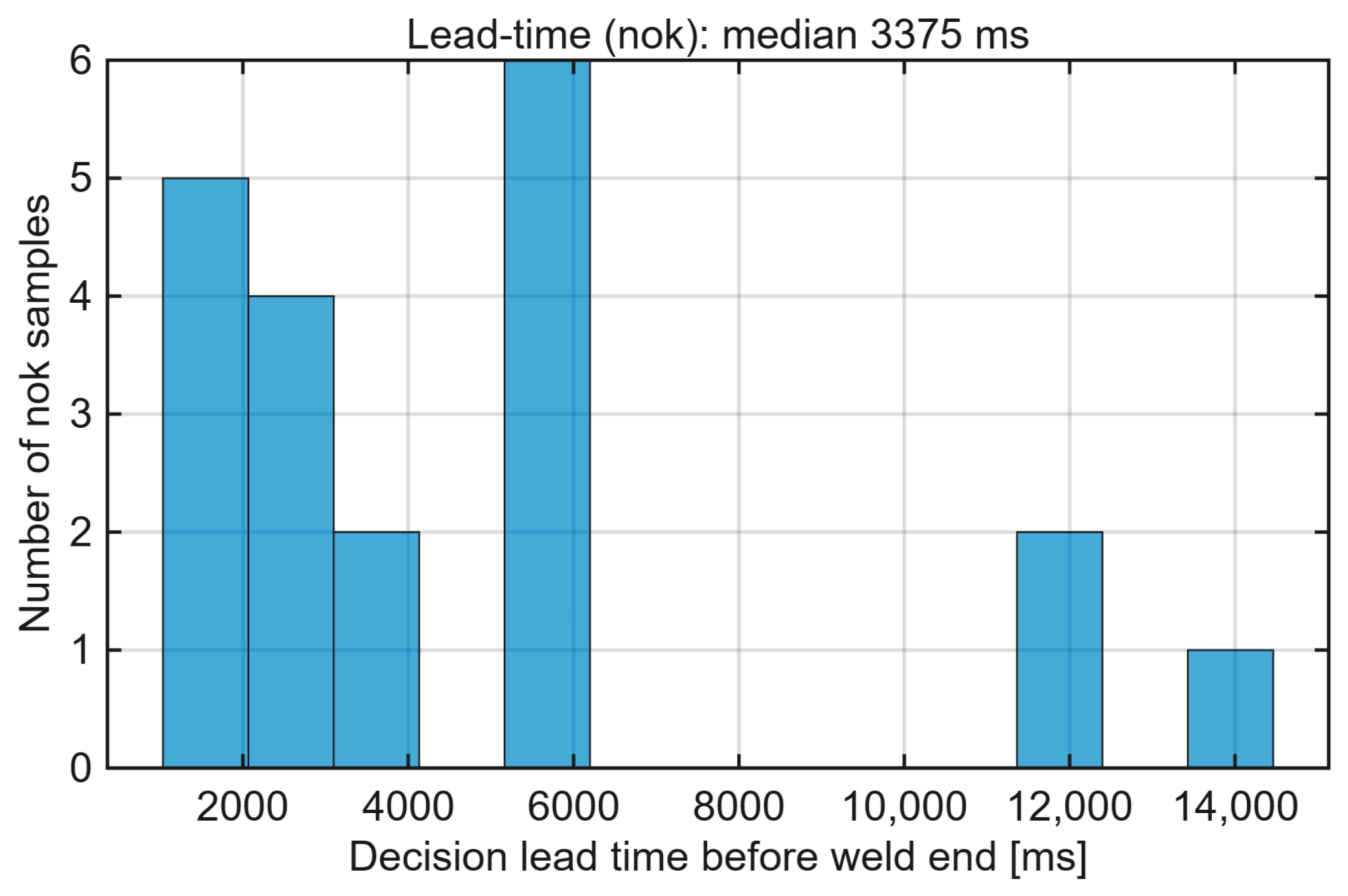
Distribution of lead time for defective welds using the online sequential rule.

Applying the sequential rule at the Youden-optimal threshold detected all 20 defective welds but produced false alarms on two of the six acceptable welds (T11 and T13), while the remaining four acceptable welds (T12 and T17–T19) were produced without an alarm. The two false alarms differed in character—T13 alarmed almost immediately (0.35 s into the pass), whereas T11 alarmed only near the end of the bead (72.1 mm, i.e., 8.65 s into an 11.55 s arc), suggesting two distinct failure modes of the rule on acceptable welds, an early transient excursion and a late accumulated drift. Decisions on defective welds were reached after only a few hundred milliseconds of arc, yielding lead times on the order of seconds (Figure 11).

In detail, 14 of the 20 defective welds were flagged after 150 ms of arc glowing, and the latest decision occurred for T10 at 700 ms, corresponding to 23.3 mm of seam at 33.33 mm/s. The resulting lead times ranged from approximately 1.2 s in the highest speed series to 13.6 s at the lowest speed. The shortest specimen T31 illustrates that lead time is bounded by the remaining bead rather than by the detector. In this case, despite an immediate decision at 150 ms, only 1.25 s of lead was available because the arc itself lasted only about 1.4 s.

### 3.5. Operating-Point Trade-Off

**Figure 12:**
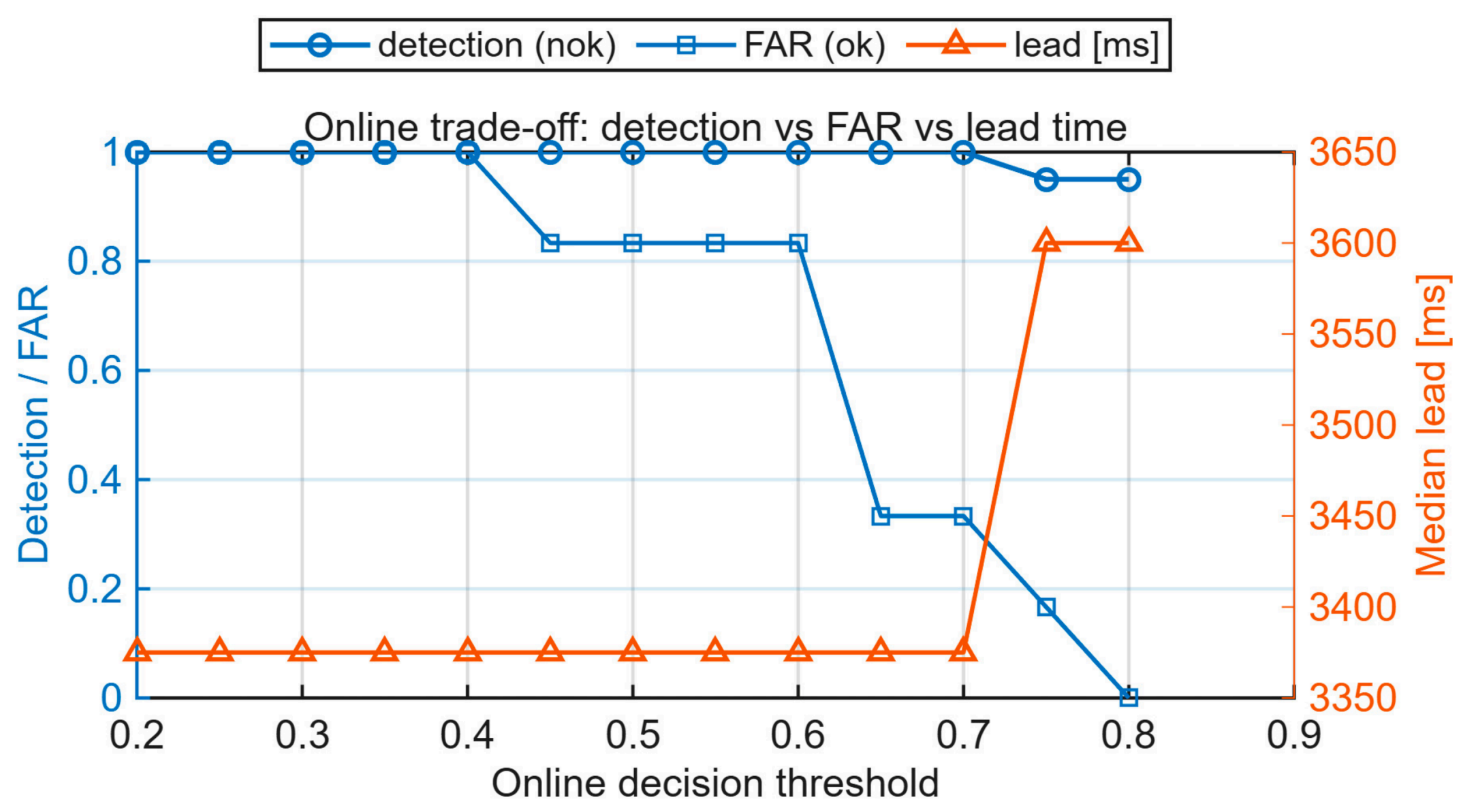
Online decision trade-off: detection rate, false-alarm rate, and median lead time versus decision threshold.

A threshold of 0.80 produced zero observed false alarms in this dataset while retaining 95% detection (one missed defective weld) and a median lead of approximately 3.6 s (Figure 12). However, because only six acceptable welds were available, each false alarm changes the estimated FAR by approximately 0.17; the specificity estimate is therefore coarse.

The structure of results presented in Figure 12 also shows that the cost of suppressing false alarms was concentrated in a narrow threshold band. Detection remained complete up to a threshold of 0.70, while the false-alarm rate fell from 1.00 to 0.33. The single lost detection appeared only at 0.75, and the last false alarm disappeared at 0.80. At the same time, the median lead varied only between approximately 3.38 s and 3.60 s across the entire sweep, which indicates that the probability trajectories of the detected defective welds saturate near 1 shortly after ignition, so the threshold setting has little influence on when the alarm fires for a true positive. In practice, this means that the strict operating point costs essentially no earliness relative to permissive settings, and the threshold can be selected on the basis of the false-alarm requirement alone.

### 3.6. Constant Decision Latency Across Welding Speed

**Figure 13:**
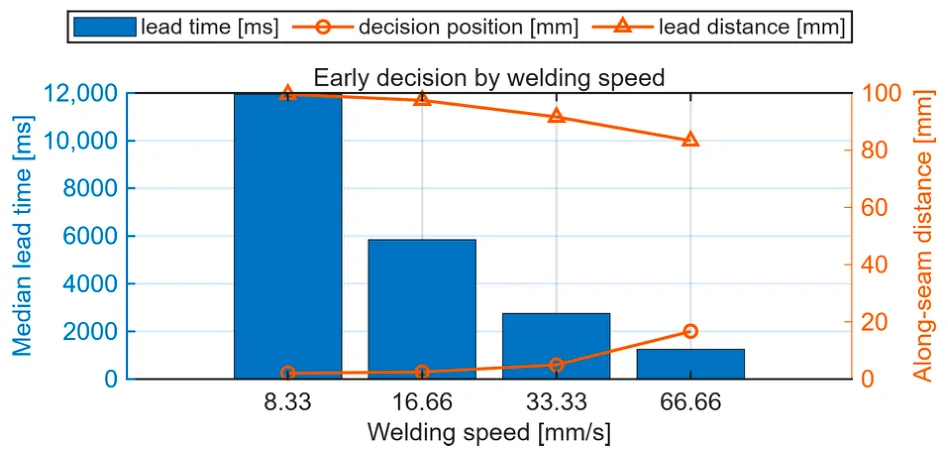
Decision position and lead time stratified by welding speed.

Decision latency was approximately constant in time (about 150–250 ms) across welding speeds, indicating that the model required a roughly fixed signal duration. This fixed time mapped to increasing spatial decision positions and decreasing lead times as welding speed increased. Even at the fastest speed, the observed warning time was approximately 1.25 s (Figure 13).

Expressed relative to the nominal 100 mm bead, the alarm consumed approximately 2% of the seam at 8.33 mm/s, 2.5% at 16.66 mm/s, 5% at 33.33 mm/s, and about 17% at 66.66 mm/s, so even in the least favorable case, more than 80% of the bead had not yet been deposited when the decision became available (median lead distance 83–100 mm; Figure 13). The two extreme speeds showed a slightly longer decision time (250 ms versus 150 ms), which may reflect either the smaller number of defective welds in those strata (*n* = 3) or genuinely slower probability saturation; in either case, the spread corresponds to only one or two window updates. The practical consequence is that a controller designed around a fixed acquisition-and-decision budget of approximately 0.25 s would remain valid across the entire tested speed range, with the remaining reaction window determined by the process rather than by the monitoring system.

### 3.7. Dependence on Process Parameters

Earliness was governed primarily by welding speed: higher speed produced a later spatial decision position and shorter lead time. Voltage had a secondary effect, whereas wire feed rate showed no significant association with lead time in this dataset.

The strong positive correlation between welding speed and decision position (ρ = +0.90) is largely a mechanical consequence of the constant-time latency documented in Section 3.6—if the decision requires an approximately fixed signal duration, then the seam length consumed before the alarm must scale with the travel speed, and the remaining lead time must shrink accordingly. Because welding speed, voltage, and wire feed rate were varied jointly across the trial series rather than in a factorial design, their individual contributions cannot be separated within this dataset. The correlations in Table 4 are therefore reported as descriptive associations only. No causal claim about voltage or wire feed is made. The absence of a wire feed effect (ρ = 0.00 for decision position) should be read together with the experimental design, in which wire feed varied jointly with voltage and travel speed across the series, so its marginal effect cannot be fully separated within this dataset.

### 3.8. Streaming Variant

**Figure 14:**
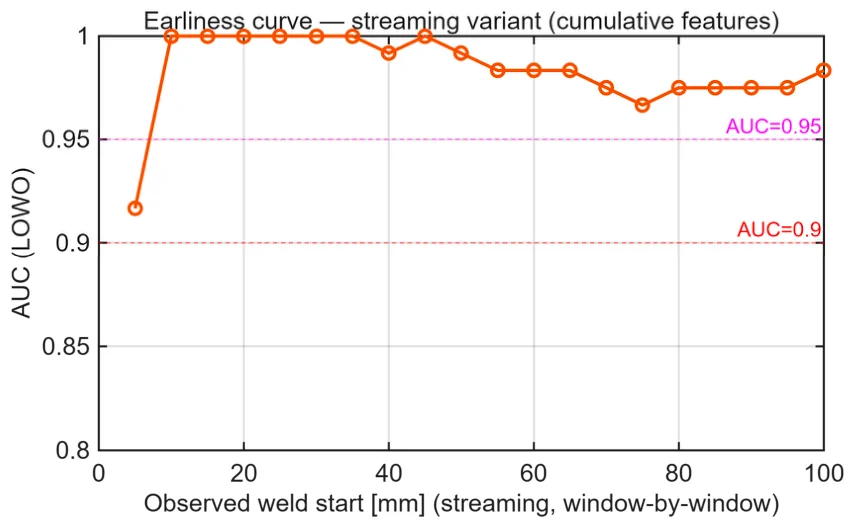
The earliness curve of the fully streaming variant.

**Figure 15:**
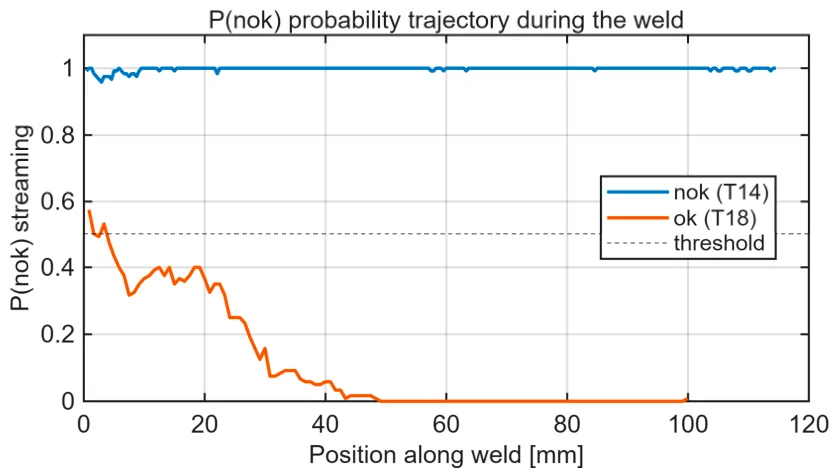
Streaming P(defective) trajectories for representative defective and acceptable welds. The blue defective-weld trajectory is drawn with increased line width to improve legibility.

The fully streaming model (Section 2.4) updated P(defective) approximately every 50 ms from cumulative causal statistics. Its earliness was sharper than the aggregate scheme (Figure 14)—AUC = 0.92 at 5 mm, while AUC = 1.00 over 10–35 mm, and again at 45 mm (0.99 at 40 mm), followed by AUC values of 0.97–0.98 up to 100 mm. Thus, a decision refreshed at each window separated acceptable from defective welds within approximately the first 10 mm and maintained very good ranking in the start and middle part of the seam (Figure 15).

The probability trajectory was, however, noisier than the aggregate score. Early cumulative statistics were unstable, so acceptable-weld trajectories occasionally exceeded the decision level. Because an alarm required only a short persistent excursion and an acceptable weld may contain hundreds of windows, the long observation horizon increased the probability of a spurious sustained excursion, a multiple-look effect. The streaming threshold sweep (Table 5) showed that the false-alarm rate could not be reduced to zero at any tested threshold: at threshold 0.50, the rule alarmed on every acceptable weld, and at threshold 0.95, FAR remained 0.20 while detection decreased to 0.76.

Two evidence-accumulating rules were tested to determine whether principled sequential detection could suppress false alarms: a one-sided CUSUM and a sequential probability ratio test (SPRT). The CUSUM statistic accumulated the excess of P(defective) above a reference level swept over {0.3, 0.4, 0.5, 0.6} with alarm limits swept over {1, 2, 3, 5, 8}, while the SPRT used target error rates alpha in {0.20, 0.10, 0.05, 0.01} with beta = 0.05 and the corresponding Wald bounds. Both rules integrate evidence over time and therefore attenuate isolated probability spikes. Their detection FAR trade-off is compared with the persistence rule in Table 6.

CUSUM and SPRT improved the middle FAR region relative to the naive persistence rule; at FAR = 0.50, both reached 0.94 detection compared with 0.88 for persistence. Nevertheless, none of the streaming rules reached zero observed false alarms. The floor is explained by two factors. First, with only six acceptable welds, a single false alarm changes FAR by approximately 0.17. Second, the random forest probabilities were over-confident, so SPRT log-likelihood increments were large, and the Wald bounds were crossed almost immediately. Probability recalibration is therefore required before SPRT thresholds can be interpreted as formal error-rate controls.

The behavior of the individual accumulating rules illustrates both the gain and its limit. For the CUSUM rule, increasing the alarm limit at a reference level of 0.3 reduced the false-alarm rate from 1.00 to 0.60 while detection remained complete. In contrast, raising the reference level to 0.5–0.6 traded detection (down to approximately 0.82) for a false-alarm rate of 0.30. None of tested settings removed false alarms entirely without simultaneously decreasing detection rate. The SPRT was almost insensitive to the target error rate. All tested values of alpha between 0.01 and 0.20 produced the same operating point (detection 0.94 at FAR 0.50), which is the expected symptom of over-confident probability inputs, because large per-window log-likelihood increments cross the Wald boundaries within a few updates regardless of where the boundaries are placed. The median lead of the streaming rules (approximately 5.5–5.9 s) exceeded that of the aggregate rule simply because their alarms typically fired within the first few windows of the trajectory.

These results separate two roles. The streaming model is an effective continuously updated early estimator, with perfect ranking over the 10–35 mm range, and evidence-accumulating sequential rules improve the alarm trade-off relative to naive persistence. However, no tested streaming rule achieved zero observed false alarms on this dataset. The aggregate prefix rule therefore remains the most robust alarming configuration, with zero observed false alarms, 95% detection, and a median lead of approximately 3.6 s at threshold 0.80.

Probability recalibration (e.g., isotonic or Platt scaling) was not applied here because reliable calibration requires more acceptable-weld data than were available; with six negatives, a calibration map cannot be fitted without reusing the same welds that define the operating point. Calibration will therefore be reserved for further research with an enlarged dataset.

### 3.9. Application to Unknown Welds

The trained model classified T20/T21/T22 as acceptable (P(defective) = 0.18–0.24) and T5/T6/T7 as borderline (P(defective) = 0.43–0.51, with T5 highest). These graded probabilities are qualitatively consistent with the expert expectation that the unknown welds were borderline. Since the same experts informed that expectation, this agreement is illustrative rather than an independent validation and is offered only to show that the rule yields a graded rather than binary early verdict.

The gradation is also consistent with the seam anomaly map of Section 3.2, in which the unknown welds occupied an intermediate band between the acceptable and defective populations. This behavior suggests a natural three-way deployment logic: probabilities near the extremes trigger automatic acceptance or an alarm, whereas the intermediate band flags the weld for targeted post-process inspection instead of an in-process intervention, so that borderline process states do not force a binary decision.

## 4. Discussion

The results show that LDV-based monitoring can support early in-process recognition of final weld state. While the baseline task addressed completed-weld detection and post-seam mapping, the central result is that the initial seam segment already contains discriminative information. The final verdict was separable from approximately the first 5 mm and perfectly separable over 30–55 mm under leave-one-weld-out validation with weld-level bootstrap confidence intervals.

This positions the proposed system as different from most published GMA welding monitoring systems. Electrical signal pipelines [5,6,7,8] and molten pool vision systems [10,11,12,13,15] typically report window-, frame-, or weld-level accuracy for completed welds, and acoustic and acoustic emission studies [16,17,18,19] have demonstrated that airborne and structural signals carry quality-relevant information, but an explicit earliness quantification—how much seam must be observed before the verdict stabilizes, together with confidence bands and a lead-time-bearing alarm—is rarely reported. The present framework is therefore complementary rather than competing—the LDV channel is independent of the arc electrical circuit, so nothing prevents fusing the prefix or streaming probability with current–voltage or optical features in installations where those sensors are already present, and the earliness-curve methodology itself is sensor-agnostic and could be applied unchanged to any window feature representation. A numerical comparison with the literature provides context, although the reported metrics and datasets are not directly comparable. Shin et al. [6] reported test accuracies of 85.8% and 89.5% for voltage-based porosity prediction. For image-based monitoring, Zhang et al. [10] reported a mean classification accuracy of 99.38%, while Li et al. [13] reported accuracy above 95%. Using air-coupled acoustic emission and welding inputs, Asif et al. [17] achieved 91.18% accuracy with sequence tagging and 82.35% with logistic regression. The present completed-weld baseline (AUC = 0.983; sensitivity = 0.95) is not directly equivalent to those accuracy values, but it indicates that non-contact torch vibration carries diagnostic information of comparable practical relevance. The distinguishing quantity is earliness—AUC = 0.94 after 5 mm and AUC = 1.00 over 30–55 mm, whereas the cited studies do not report how much of the joint must be observed before the verdict stabilizes.

The sequential rule converted the probability stream into an actionable alarm with a measurable and acceptable time margin. At the operating point with no observed false alarms (threshold 0.80), it retained 95% detection with a median lead of approximately 3.6 s. The streaming variant provided a verdict refreshed approximately every 50 ms, which is the natural interface to a controller, but it required additional calibration to avoid streaming false alarms.

A key deployment finding is that decision latency was approximately constant in time. A fixed acquisition buffer of roughly 0.15–0.25 s was sufficient across the tested speeds, but the spatial decision position and remaining lead time changed with speed. This explains why the available reaction window decreased from approximately 12 s at 8.3 mm/s to approximately 1.25 s at 66.7 mm/s. It should also be noted that industrial deployment of a laser Doppler vibrometer brings its own practical constraints, such as sensor cost, beam alignment, and sensitivity of the measurement to surface condition and spatter contamination of the optics; these aspects were not limiting in the laboratory setup, but they must be verified in production conditions before the sensor-light claim can be extended to the shop floor.

It is also worth translating the reported lead times into actionable margins. At intermediate speeds, the alarm was available after 2.5–5 mm of a nominally 100 mm bead, i.e., with roughly 95% of the bead still to be deposited, and the median lead of approximately 3.6 s is long relative to the reaction time of a modern inverter power source and the command cycle of a welding robot, both of which operate on the millisecond scale. The margin is therefore sufficient in principle for parameter correction, controlled termination of the pass, or marking of the part for selective rework. The binding constraint in deployment is consequently not the monitoring latency, but the design of a safe intervention policy—what the controller should actually do upon an alarm—which lies outside the scope of the present study.

The parameter dependence is physically interpretable. Welding speed determines how much seam length elapses during the fixed decision latency, and voltage modulates arc stability reflected in the vibration signature. The absence of a significant wire feed effect in this dataset may reflect the limited design space and should not be generalized beyond the tested conditions.

A physical reading of the early separability is consistent with the destabilization mechanisms used in the experiment. Both instability triggers—short-circuiting of the wire against the solid front of the pool at excessive travel speed and irregular transfer at reduced voltage—act from the first short-circuit cycles after ignition, and each rupture and re-ignition delivers an impulsive excitation to the torch that the vibrometer observes directly at the gas nozzle. Because the defective welds in this dataset were destabilized over their entire length, the discriminative signature is present essentially from arc ignition, which explains why 5 mm of observed seam already yielded AUC = 0.94 and why the ignition transient around 10 mm reduced, rather than created, separability. The same reasoning delimits the claim—for faults that develop gradually in mid-weld, the earliness observed here cannot be assumed, and the earliness curve would have to be re-estimated on data containing genuine state transitions. Specimen T33 behaves exactly as this reasoning predicts. Its process is stable over approximately the first 74 mm, so no causal prefix model can classify it correctly from the initial millimeters, and it is indeed missed at the shortest observed lengths; the window-level anomaly index, which does not aggregate over the prefix, nevertheless marks the transition at the position where it physically occurs. The earliness curve reported here is therefore a lower bound for established-state recognition and should not be read as an estimate of how early a mid-weld onset could be anticipated.

The completed-weld detection and seam mapping are included only as a baseline that verifies the diagnostic value of the LDV channel. The novel contribution of this manuscript is the causal early-recognition framework: the earliness curve with confidence bands, streaming probability trajectories, sequential alarm with lead time, speed-stratified latency analysis, and parameter-dependence study.

This study has five important limitations. First, only six acceptable welds were available, so specificity and FAR estimates are coarse, and one-class AUC values of 1.00 are optimistic. Second, defective beads were mostly defective along their entire length. The task is therefore early recognition of an already defective process state rather than anticipation of a good-to-defective transition; lead time denotes how soon the established state was recognized. The single exception is T33, in which the process remained stable over approximately the first 74 mm. This specimen cannot be recognized early by construction, and it is among the welds missed by the prefix model at the shortest observed lengths, while the seam anomaly map localizes its transition correctly (Section 3.2); it therefore delimits the earliness result rather than contradicting it. Third, one defective weld (T23) was missed at the zero-observed-false-alarm operating point, and one specimen (T31) was genuinely short. Fourth, streaming sequential rules produced false alarms because of multiple testing over many windows and the small acceptable-weld set. Fifth, all specimens were bead-on-plate deposits, so the influence of the joint geometry itself—root gap variation, sidewall heat extraction, restraint, and multi-pass thermal history—is not covered by the present results; locally acting disturbances of this kind may produce short signatures that a cumulative prefix representation partly averages out, and the transferability of the trained model to grooved and fillet joints therefore has to be re-verified experimentally. A larger, balanced dataset with deliberately introduced transitions in various parts of weld is required to support stronger claims about defect-onset anticipation and to extend the method to the more general and complex cases that may occur in industrial applications.

## 5. Conclusions

This study examined whether the final quality state of a GMA weld can be recognized during welding from the initial segment of the seam, using a laser Doppler vibrometer as the sole diagnostic sensor. Under leave-one-weld-out validation with weld-level bootstrap confidence intervals, the final verdict was separable from the first 5 mm of observed seam (AUC = 0.94) and perfectly separable over 30–55 mm (AUC = 1.00). Non-contact measurement of torch vibration therefore carries sufficient information for early recognition of an unstable GMA process, without access to the welding current as a diagnostic input.

Converted into an online alarm, the aggregate prefix rule reached an operating point with zero observed false alarms on the available acceptable welds, 95% detection, and a median lead time of approximately 3.6 s at a decision threshold of 0.80. Decision latency was approximately constant in time (0.15–0.25 s) across the tested welding speeds, so the spatial decision position and the remaining lead time were governed mainly by the welding speed; voltage showed a moderate secondary effect, whereas wire feed showed no significant effect within the tested parameter range. The fully streaming variant additionally provided a probability of a defective weld refreshed approximately every 50 ms with near-perfect early ranking, but it requires probability recalibration and a larger acceptable-weld set before its sequential alarms can operate without false alarms.

Taken together, with the explicit reservation that the specificity and false-alarm estimates rest on a small and imbalanced dataset in which the defective welds were, with a single exception, defective along their full length, the proposed method gave promising results. Future work will therefore enlarge the balanced dataset, introduce controlled mid-weld transitions to test genuine defect-onset anticipation, recalibrate the streaming probabilities, integrate the predictor with a parameter controller, and validate the method across materials, joint geometries, and production environments.

## Figures and Tables

**Table 1:** Welding parameter sets used for the bead-on-plate trials. Voltage and wire feed are the values adjusted on the power source. Welding current was measured during each pass. The last two columns give the mean welding current of the set and the standard deviation of the current within a pass, both averaged over the passes belonging to the set.

Designators	Set Voltage [V]	Wire Feed [m/min]	Welding Speed [cm/min]	Measured Current [A]	Within-Pass SD [A]
T5–T7	18	4	100	133.08	3.62
T8–T10	18	4	200	134.89	5.19
T11–T13	18	4	50	130.95	3.71
T14–T16	12	4	50	161.20	28.50
T17–T19	21	8	100	215.82	3.63
T20–T22	21	8	200	218.48	4.68
T23–T25	21	8	400	218.01	5.11
T26–T28	12	8	200	245.52	26.26
T29–T32	12	8	100	263.79	16.31
T33–T36	16	8	100	234.91	16.43

**Table 2:** Feature families extracted from each 0.10 s LDV window (38 features in total).

Feature Family	Count	Features (Abbreviations)
Time domain	12	RMS, Peak, P2P, Std, Skew, Kurt, Crest, Shape, Impulse, Clearance, ZCR, Energy
Frequency domain	15	SpecCentroid, SpecSpread, SpecSkew, SpecKurt, SpecEntropy, SpecRolloff, FreqPeak, SpecFlatness, TotPower, BandRatio1–6
Envelope (Hilbert)	3	EnvRMS, EnvKurt, EnvModFreq
Wavelet (MODWPT, level-3)	8	WPEnergy1–8

**Table 3:** Causal early prediction: AUC (95% bootstrap CI), accuracy, sensitivity, and specificity versus observed start length L (leave-one-weld-out strategy).

L [mm]	AUC	95% CI	Accuracy	Sensitivity	Specificity
5	0.942	[0.82, 1.00]	0.923	0.90	1.00
10	0.850	[0.65, 0.99]	0.769	0.75	0.83
15	0.967	[0.86, 1.00]	0.962	1.00	0.83
20	0.933	[0.73, 1.00]	0.962	1.00	0.83
25	0.967	[0.86, 1.00]	0.962	1.00	0.83
30–55	1.000	[1.00, 1.00]	1.000	1.00	1.00
60–70	0.992	[0.94, 1.00]	0.962	0.95	1.00
80	0.958	[0.86, 1.00]	0.962	0.95	1.00
90	0.925	[0.80, 1.00]	0.846	0.80	1.00
100	0.933	[0.80, 1.00]	0.885	0.85	1.00

**Table 4:** Spearman correlation of online decision metrics with process parameters (defective welds).

Parameter	ρ (Decision Position)	*p*	ρ (Lead Time)	*p*
Weld speed	+0.90	9.0 × 10^−8^	−0.86	1.2 × 10^−6^
Voltage U	+0.64	0.0023	−0.53	0.017
Wire feed	0.00	1.00	−0.30	0.19

**Table 5:** Performance of the streaming persistence rule across decision thresholds.

Threshold	Detection (Defective)	FAR (Acceptable)	Median Lead [ms]
0.30–0.50	1.00	1.00	5850
0.55	1.00	0.80	5850
0.65–0.70	1.00	0.60	5850
0.80	0.88	0.40	5800
0.90	0.82	0.30	5600
0.95	0.76	0.20	5550

**Table 6:** Rules for online streaming—detection at matched false-alarm rates (best per rule).

FAR (Acceptable)	Persistence Detection	CUSUM Detection	SPRT Detection
0.60	1.00	1.00	-
0.50	0.88	0.94	0.94
0.40	0.88	0.88	-
0.30	0.82	0.82	-
0.20	0.76	-	-
0.60	1.00	1.00	-

## Data Availability

The data presented in this study are available from the corresponding author upon reasonable request.

## Abbreviations

The following abbreviations are used in this manuscript:

AUC	Area Under the Curve
CI	Confidence Interval
CNN	Convolutional Neural Network
CUSUM	Cumulative Sum
FAR	False-Alarm Rate
GMA	Gas Metal Arc
GMAW-S	Gas Metal Arc Welding—Short-circuiting
IQR	Interquartile Range
LDV	Laser Doppler Vibrometry/Vibrometer
LSTM	Long Short-Term Memory
MAD	Median Absolute Deviation
MAG	Metal Active Gas
MODWPT	Maximal Overlap Discrete Wavelet Packet Transform
NDT	Non-Destructive Testing
PCA	Principal Component Analysis
RBF	Radial Basis Function
RMS	Root Mean Square
SPRT	Sequential Probability Ratio Test
SVM	Support Vector Machine
